# Analyzing the microRNA Transcriptome in Plants Using Deep Sequencing Data

**DOI:** 10.3390/biology1020297

**Published:** 2012-08-15

**Authors:** Xiaozeng Yang, Lei Li

**Affiliations:** Department of Biology, University of Virginia, Charlottesville VA 22904, USA; Email: xy2y@virginia.edu

**Keywords:** microRNA, miRDeep-P, next-generation sequencing, small RNA, plant

## Abstract

MicroRNAs (miRNAs) are 20- to 24-nucleotide endogenous small RNA molecules emerging as an important class of sequence-specific, *trans*-acting regulators for modulating gene expression at the post-transcription level. There has been a surge of interest in the past decade in identifying miRNAs and profiling their expression pattern using various experimental approaches. In particular, ultra-deep sampling of specifically prepared low-molecular-weight RNA libraries based on next-generation sequencing technologies has been used successfully in diverse species. The challenge now is to effectively deconvolute the complex sequencing data to provide comprehensive and reliable information on the miRNAs, miRNA precursors, and expression profile of miRNA genes. Here we review the recently developed computational tools and their applications in profiling the miRNA transcriptomes, with an emphasis on the model plant *Arabidopsis thaliana*. Highlighted is also progress and insight into miRNA biology derived from analyzing available deep sequencing data.

## 1. Introduction

One of the most exciting biological finding in recent years is the discovery of many functional small RNA species that regulate diverse spatial and temporal function of the genome [1,2,3,4]. After the initial discovery of miRNAs in the worm *C. elegans* [5,6], they are emerging as an important class of endogenous gene regulators acting at the post-transcriptional level in both animals and plants. In plants, much of the effort to identify, experimentally validate, and functionally characterize miRNAs has been directed toward the model plant *Arabidopsis thaliana*. Consequently, dozens of miRNA‑target pairs have been identified and studied [7,8,9]. It is now well-established that these gene circuits are crucial for many plant development processes as well as responses to environmental challenges [4,9,10].

**Figure 1:**
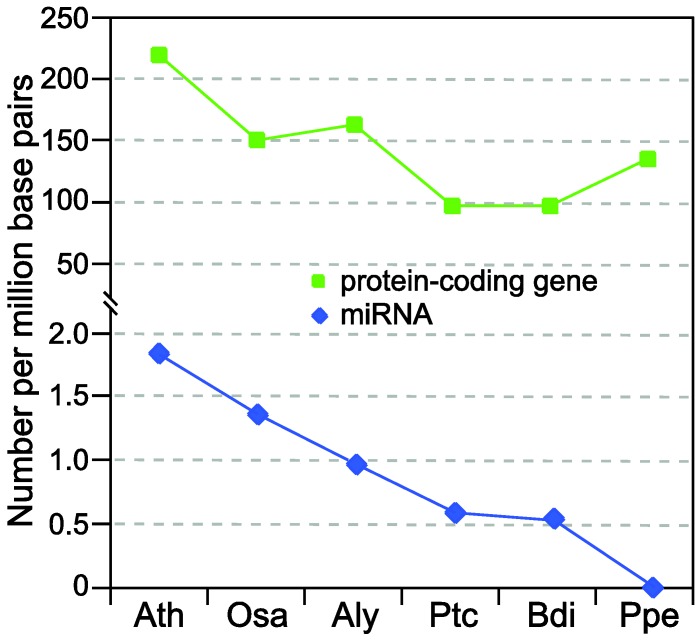
Comparison of the density of protein-coding and *MIR* genes in six plant lineages. Vertical axis indicates the density of protein-coding and *MIR* genes per million genomic base pairs. The six plants are Ath (*Arabidopsis thaliana*), Osa (*Oryza sativa*), Aly (*Arabidopsis lyrata*), Ptc (*Populus trichocarpa*), Bdi (*Brachypodium distachyon*) and Ppe (*Prunus persica*). The numbers of protein-coding genes are obtained from TAIR10 (Ath), RGAP6.1 (Osa) [12] and Phytozome7.0 (Aly, Ptc, Bdi and Ppe) [13] while all the numbers of *MIR* genes are from miRBase17 [11].

Although hundreds of miRNAs have been predicted in a broad range of plant lineages [11], there are two indications that current miRNA collections in many model plants and important crop species are far from completion. First of all, the numbers of predicted miRNAs among different plant species are conspicuously uneven. As shown in Figure 1, the well-annotated *Arabidopsis* and rice (*Oryza sativa*) genomes contain approximately one miRNA for every 100 protein-coding genes. In other plant species, the relative density of miRNAs is only half or even less than that in *Arabidopsis* and rice (Figure 1). Because it is highly unlikely that these species indeed encode a smaller complement of miRNA genes, the only explanation is that most miRNAs in species other than *Arabidopsis* and rice still await discovery.

In plants, various studies have established that there are about 20 families of conserved miRNAs [14,15]. In many plant species, only miRNAs belonging to conserved families are identified. However, the sequencing of small RNA populations is increasingly revealing miRNAs that are not conserved between species, suggesting a recent evolutionary origin [8,14,16,17,18]. In fact, there is increasing evidence that species-specific or subfamily-specific miRNAs are functional constituents of the miRNA-mediated regulatory networks and underscore the dynamic nature of these networks [19,20,21]. Thus, it is highly desirable to elucidate the full spectrum of miRNAs in diverse plant lineages to gain a comprehensive understanding of miRNA origin, evolution and function. 

## 2. Brief Overview of miRNA Biogenesis in Plants

**Figure 2:**
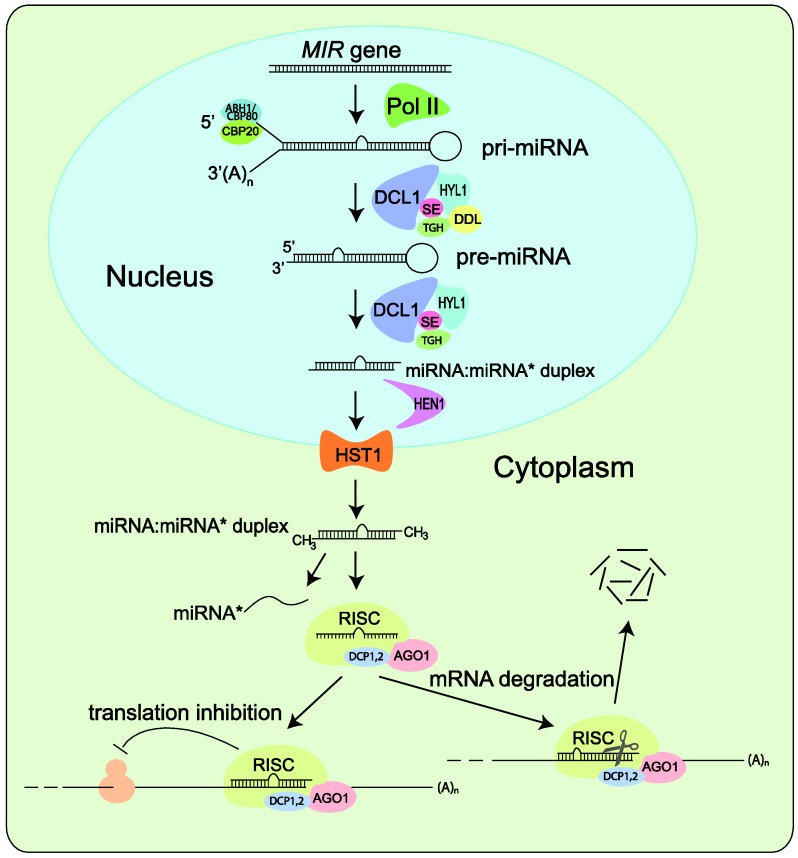
Simplified model of miRNA biogenesis in plants. *MIR* genes are initially transcribed by Pol II into pri-miRNAs that fold back to form hairpin structure. Splicing and further processing in nuclear involve the interactive functions of HYL1, DDL, TGH and SE and of the cap-binding proteins CBP20 and CBP80. Pri-miRNAs and pre-miRNAs are sequentially processed by DCL1 to yield one or several phased miRNA/miRNA* duplexes, which are methylated by HEN1 and transported to the cytoplasm by HST1. The miRNA is selected and incorporated into dedicated AGO1-containing RISC that directs translation inhibition or cleavage of the target mRNA transcript.

There is no question that any systematic effort to identify miRNAs will depend on a clear understanding of the miRNA biogenesis pathways. Since miRNA biogenesis is the subject of numerous reviews, we only provide a brief overview here (Figure 2). Like protein-coding genes, miRNAs are encoded by class II genes and transcribed by RNA polymerase II [22,23]. Although mature miRNAs are typically 20- to 24-nucleotides (nt) in length, their precursor transcripts can be much longer. As shown in Figure 2, after initial transcription by Pol II, splicing and further processing of pri-miRNAs are carried out in the nucleus and involve the interactive functions of HYL1, DDL, TGHand SE, as well as the cap-binding proteins CBP20 and CBP80 [24,25,26]. 

A characteristic of pri-miRNAs is that they contain internally complementary sequences that fold back to form a hairpin structure, which is called pre-miRNAs [2,4,27,28]. Pri-miRNAs and pre‑miRNAs are sequentially processed by Dicer to yield one or several phased miRNA/miRNA* duplexes. Unique to higher plants, pri-miRNA and pre-miRNA processing are both carried out in the nucleus [29]. The duplexes are stabilized through end methylation catalyzed by HEN1 [25] and transported to the cytoplasm by HST1 [26]. Only the mature miRNA is integrated into the AGO1‑containing RNA-induced silencing complex (RISC) and accumulated with DCP1 and DCP2 whereas the passenger strand, called RNA*, is degraded as a RISC substrate [2,30,31,32]. After loading into RISC, miRNAs base pair with their targets and direct either cleavage [33,34] or translational repression [35] of the target transcripts. Recently, silencing of target genes by miRNA‑directed DNA methylation at the target loci has also been reported [36]. 

As a consequence of miRNA maturation, a series of RNA intermediates are generated in addition to the mature miRNAs, which include the stem-loop-structured pre-miRNAs, miRNA* and sliced RNA fragments derived from other parts of the precursors. Detection, quantification, and reconstruction of these RNA intermediates are the goal of essentially all available methods to identify and profile miRNAs.

## 3. Comprehensive Identification of miRNAs from Next-Generation Sequencing Data

Direct sequencing of specifically prepared low-molecular-weight RNA has long been recognized as powerful approach to sample the small RNA species [37]. Typically, small RNA species in the 18–35 nucleotides range are isolated and ligated to the 5' and 3' RNA adapters. The ligated RNA molecules are reverse-transcribed into cDNA using a primer specific to the 5' adapter and amplified by PCR with two primers that anneal to the ends of the adapters. Quality controlled cDNA libraries are then sequenced [8,37]. In one of the earliest studies, Fahlgren *et al.* [8] sequenced small RNA populations from wild-type Arabidopsis as well several mutants defective in miRNA biogenesis using the 454 technology. A total of 48 non-conserved miRNA families were identified by a computational analysis of sequence composition and secondary structure based on knowledge of annotated miRNAs at that time [8].

Encouraged by success in *Arabidopsis* [8,17], deep sampling of small RNA libraries by next‑generation sequencing has become a popular approach to identify miRNAs for functional and evolutionary studies in diverse plant species [18,38,39]. An advantage of the sequencing methods is their sensitivity in detecting even poorly expressed or species-specific miRNAs [39]. The potential of deep sequencing to provide quantitative information on the expression pattern of known miRNAs has been explored [8,40]. In addition to validating annotated miRNAs, large numbers of putative new miRNAs have been identified. Following we discuss various recently developed algorithms and programs to profile miRNAs from deep sequencing data, with an emphasis on their applications in plants. 

## 4. Available Tools for Analyzing miRNAs from Deep Sequencing Data

A number of computational tools for identifying and profiling miRNAs from deep sequencing data have been developed. With an easy-to-use graphical interface, most of these tools are web-based while a few, such as miRDeep [41] and miRNAKey [42], are also packaged into a stand-alone version (Table 1). A common module employed by these tools is sequence similarity search to detect miRNAs cross multiple species based on the fact that many miRNAs are evolutionarily conserved. Meanwhile, other algorithms are also introduced to detect new miRNAs based on different models in terms of the pre-miRNA hairpin structures and the duplex of miRNA and miRNA* (Table 1). The challenge now is to separate miRNAs from the pool of other sequenced small RNAs or mRNA degradation products. Further, as most of the miRNA-detecting methods focus exclusively on the mature miRNAs, a drawback is the failure to collect and quantify information on the precursors, which could result in limitations in elucidating the miRNA transcriptome.

**Table 1:** Tools for analyzing deeply sequenced small RNA data.

Name	Designed Model	Algorithm	Availability
UEA sRNA Toolkit ^*^	animal & plant	based on criteria of miRNAs	web-based
miRDeep	animal	probabilistic model	stand-alone
miRanalyzer ^**^	animal	machine learning	web-based
SeqBuster [43]	animal	sequence similarity	web-based
DSAP [44]	animal	sequence similarity	web-based
mirTools [45]	animal	sequence similarity & miRDeep	web-based
miRNAKey	animal	sequence similarity	stand-alone
miRNEST	animal & plant	sequence similarity	web-based

* Only the miRCat component is for detecting new miRNAs; ** Updated miRanalyzer could also predict new miRNAs in plants, and it has a new stand-alone version.

### 4.1. miRanalyzer

Utilizing a machine learning algorithm, Hackenberg *et al.* [46] developed miRanalyzer, a web server tool for analyzing results from deep-sequencing experiments on small RNAs. This program requires a simple input file containing a list of unique reads and their copy numbers. Application of this program in seven animal model species (human, mouse, rat, fruit fly, round-worm, zebra fish and dog) not only detected known miRNA sequences annotated in miRBase, but led to prediction of new miRNAs. The core algorithm of miRanalyzer is based on the *random forest* classifier and was trained on experimental data, which could accurately predict novel miRNAs with a low false positive rate in animals. Later, miRanalyzer was updated to include a module on miRNA prediction in plants by taking into account differences between plant and animal miRNAs. Currently, 31 genomes, including 6 plant genomes, have been analyzed by the updated miRanalyzer [47]. 

### 4.2. UEA sRNA Toolkit

UEA sRNA Toolkit [48] combines two integrated parts, miRCat and SiLoCo, to analyze miRNAs using deep sequencing data. miRCat, the package for detecting miRNAs, adopts a number of empirical and published criteria for *bona fide* miRNA loci to mine miRNAs from deeply sequenced small RNA data. In brief, the program accepts a FASTA file of small RNA sequences as input, which are mapped to a plant genome using PatMaN [49] and grouped into discrete loci. Then it obtains miRNA candidates by searching for a two-peak alignment pattern of sequence reads on one strand of the locus and assessing the secondary structures of a series of putative precursor transcripts using RNAfold [50] and randfold [51]. On the other hand, SiLoCo is the tool to compare the miRNA expression between different samples. It weighs each small RNA hit by its repetitiveness in the genome acquired from mapping by PatMaN [49]. For each locus, the log2 ratio and the average of the normalized small RNA hit counts are used to calculate the miRNA expression difference [48].

### 4.3. miRDeep

**Figure 3:**
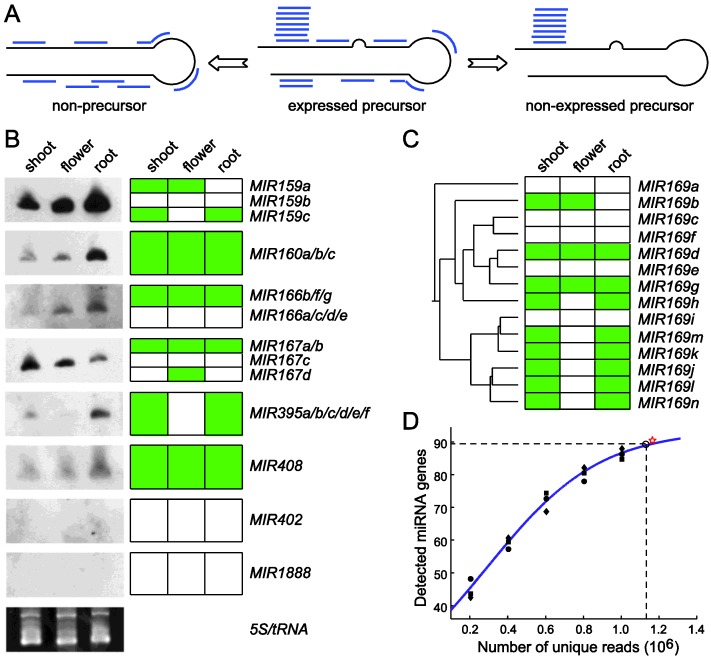
Using miRDeep-P to identify and profile miRNAs from deep sequencing data. (**A**) The core algorithm is based on a miRNA biogenesis model in which the small RNAs derived from a pre-miRNA are considered to have certain probabilities of being sequenced [41]. This model distinguishes an expressed pre-miRNA from a non-expressed pre-miRNA or a genomic locus with the potential to form a hairpin but is not processed by Dicer. (**B**) Validation of miRDeep-P results in Arabidopsis by Northern blotting. RNA blots are shown on the left. Ethidium bromide staining of the 5S/tRNA is used as a loading control. The expression pattern (represented by the green color) of individual genes deduced from the sequencing data is shown on the right with genes with identical pattern combined. (**C**) Relating miRNA expression to pre-miRNA phylogeny. The annotated pre‑miRNAs of the miR169 family in Arabidopsis were used to construct a phylogenetic tree. The gene‑level expression profile of family members is depicted to the right of the tree. (**D**) Simulation of the relation between miRNA detection rate and sequencing depth in Arabidopsis. Perfectly mapped unique reads from the shoot library were randomly retrieved to create five different simulated sequencing depths. The number of expressed miRNAs was determined at each depth. The scatter plot represents results from three independent simulations and was used for curve fitting. The star sign indicates the actual data from the shoot library. Dashed lines indicate a 95% detection rate of the theoretic maximal number of expressed miRNAs and the corresponding sequencing depth. Adapted from Yang *et al.* [52].

Maturation of miRNAs from the stem-loop structured pre-miRNAs results in three species of small RNAs: mature miRNA, miRNA* and RNA fragments derived from other parts of the precursors. Typically the mature miRNAs are very stable, which results in uneven abundance of the different small RNA species derived from the same pre-miRNA (Figure 3A). miRDeep, developed by Friedländer *et al.*, employs a novel algorithm based on a probabilistic model of miRNA biogenesis to score compatibility of the nucleotide position and frequency of sequenced small RNA reads with the secondary structure of miRNA precursors [41]. When using miRDeep, the small RNA sequence reads are first aligned to the genome. The genomic DNA bracketing these alignments are extracted and computed for secondary RNA structure. Plausible miRNA precursor sequences based on a model for Dicer-mediated miRNA processing are identified. Finally, miRDeep scores the likelihood of the putative miRNA precursors and outputs a scored list of known and new miRNA precursors and mature miRNAs in the deep-sequencing samples [41]. 

### 4.4. miRNAKey

miRNAkey [36] is a software pipeline designed to be used as a base-station for the analysis of deep sequencing data on miRNAs. The package implements a common set of steps generally taken to analyze deep sequencing data including the use of similarity search to detect known or conserved miRNAs as well as adding miRDeep [41] to predict new miRNAs. This program also includes unique features such as data statistics and multiple mapping levels to generate a comprehensive platform for the analysis of miRNA expression. Based on a statistic analysis of small RNA sequencing data, it could generate measurement of differentially expressed miRNAs in paired samples by a tabular and graphical output format.

It should be noted that most of the programs listed in Table 1 are originally designed for analyzing miRNAs in animals. Several considerations prevent their direct application to profile miRNAs in plants. First, many available methods highly depend on sequence similarity search to detect known and new miRNAs, which is not sufficient to uncover species-specific miRNAs. In fact, only a minority of annotated miRNAs in plants is conserved between different lineages, suggesting that most unknown miRNAs would not be discovered through sequence similarity search [14]. Second, for those programs that do consider other features their models are usually based on the animal systems. However, it is well studied that some aspects of miRNA biogenesis in plants and animals are critically different. For instance, pre-miRNAs in animals possess a rather uniform length at ~80 nucleotides, which is a key to the success of miRDeep, miRCat, and miRNAKey [41,42,48]. In plants, the precursor length is longer and more variable. Thus, it is not feasible to simply employ tools developed for animals to detect plant miRNAs. Third, considering the easy-to-use feature, most tools in Table 1 are available as a web-based version, which could easily handle small size sequencing data. However, at present it is standard to generate tens of millions of reads per sample, resulting in increased difficulty of processing the large amount of data on line and reinforcing the desirability of developing plant-specific tools.

## 5. miRDeep-P Is a Program for Comprehensive Identification of miRNAs in Plants

miRDeep-P was modified from miRDeep [41] to specifically retrieve and quantify miRNA related information from deep sequencing data in plants [52,53]. Similar to miRDeep, this program maps the small RNA reads to a reference genome and extracts the sequence flanking each anchored read for predicting RNA secondary structure and quantifying the compatibility of the distribution of reads with Dicer-mediated processing. After progressively processing all mapped reads, candidate miRNAs as well pre-miRNAs are scored based on the core miRDeep algorithm [41] and filtered with plant‑specific criteria [52,53,54]. It thus provides reliable information on the transcription and processing of the pre-miRNAs (Figure 3A). Using training data from both Arabidopsis and rice, it was demonstrated that miRDeep-P works effectively for deep sequencing data in plants [52]. miRDeep‑P is freely available as a stand-alone package that runs in a command line environment [53]. 

miRDeep-P was tested utilizing annotated miRNAs in *Arabidopsis* and available deep sequencing data from three independent small RNA libraries prepared from shoot, root and inflorescence [52]. By retrieving the signature small RNA distribution from each of the 199 annotated pre-miRNAs (miRBase release 15), the tissue-specific expression pattern of individual miRNAs was determined. In shoot, root and flower, 81, 70 and 55 expressed pre-miRNAs were detected, respectively, indicating that only 40% of the annotated pre-miRNAs are expressed in major organ types. Northern blotting was performed and the results were clearly consistent with expression determined from the deep sequencing data for miRNAs of single member families. For the multiple member families, the cumulated expression levels combining individual gene-level expression also showed strong agreement with that from the Northern analysis (Figure 3B). 

Gene specific expression patterns generated by miRDeep-P revealed the transcriptional relationship of paralogous members. For example, the *MIR169* family has 14 members in *Arabidopsis*. According to the neighbor-joining tree constructed from pre-miRNA sequences, this family could be grouped into three major clusters (Figure 3C). The smallest clade only consisting of *MIR169a* was not expressed according to miRDeep-P analysis. By contrast, the clade consisting of *MIR169i/j/k/l/m/n* was expressed simultaneously but only in root and shoot. The only exception for this clade was *MIR169i*, which was not expressed. Meanwhile, the *MIR169b/c/d/e/g/f/h* branch was detected in flower as well (Figure 3C). These results indicate that the tissue specific expression determined from deep sequencing data by miRDeep-P is consistent with the phylogenetic relationship of paralogous *MIR* genes. 

Reliable estimation of gene level miRNA expression makes it possible to determine the relationship between miRNA detection rate and the sequencing depth. A simulation approach was taken in which sequence reads in the shoot library were randomly selected to simulate six different sequencing depths. These subsets were processed using miRDeep-P and the number of expressed miRNAs at each sequencing depth calculated. After reiterating this process for three times, the mathematical relationship between the number of detected miRNAs and the number of unique sequence reads was determined by curve fitting based on a logistic function (Figure 3D). This analysis indicates that, when sampling of the RNA population is unbiased, there would be a finite number of reads needed to reach a saturated detection rate of expressed miRNAs. Further, the maximal number of expressed miRNAs in shoot was estimated to be 94. Accordingly, 1.13 million unique, perfectly mapped sequence reads were required to detect 95% of the expressed miRNAs in this simulation (Figure 3D).

## 6. Plant miRNA Databases with Integrated Deep Sequencing Data

Increasing accumulation and mining of deep sequencing data have resulted in the development of comprehensive databases to facilitate miRNA annotation in a variety of species or experimental systems. The earliest and most comprehensive miRNA database, miRBase, combines deep sequencing data with miRNA annotation in chromalveolata, metazoan, mycetozoa, viridiplantae, and viruses [55]. On the other hand, genome annotation databases for plant species such as TAIR and TFGD [56] also include miRNA loci. The online databases most relevant to plant miRNAs are summarized in Table 2. ASRP, the Arabidopsis Small RNA Project Database, is the first database providing a repository for sequences of miRNAs from various *Arabidopsis* genotypes and tissues [57]. PmiRKB, currently focusing on the two model plants *Arabidopsis* and rice was developed to emphasize on single nucleotide polymorphisms regarding miRNAs in these two species supported by deep sequencing data [58]. miRNEST is a newly released comprehensive miRNA database including miRNA sequences from more than 200 plants, and those annotated miRNAs from some model plants are supported by deep sequencing data from different samples [59]. 

Transcriptome profiling has become indispensable in biology, which now includes not only mRNA but also other regulatory RNA species and intermediates of RNA metabolism. To fully decipher the transcriptome, systems based approaches are highly desirable to integrate the expression profiles with data characterizing other functional elements of the cell. Toward this goal, databases integrating miRNA annotation and deep sequencing data represent an important step forward. Combining sequencing data from various genotypes, diverse tissues, different developmental stages, or in response to different environmental challenges, could elaborate the expression patterns of miRNAs. Further, these databases should prove useful to integrate deep sequencing data of small RNA with other types of high throughput data such as those for mRNA transcriptome and degradome. The integrated data, in conjugation with modeling and model testing, will provide important clues to the regulatory networks that ultimately elucidate genome transcription, function, and adaptation. 

**Table 2:** Databases for plant miRNAs with integrated deep sequencing data.

Database	Description	URL
miRBase	Including miRNA sequences and annotations in more than 50 plants.	http://www.mirbase.org/
miRNEST	Combining miRNA sequences in more than 200 plant organisms.	http://lemur.amu.edu.pl/share/php/mirnest/home.php
PmiRKB	*Arabidopsis* and Rice miRNA knowledge base.	http://bis.zju.edu.cn/pmirkb/
ASRP	Database of *Arabidopsis* small RNA sequences.	http://asrp.cgrb.oregonstate.edu/
